# Spondylolysis in Young Athletes: An Overview Emphasizing Nonoperative Management

**DOI:** 10.1155/2020/9235958

**Published:** 2020-01-21

**Authors:** Sara Goetzinger, Selen Courtney, Kathy Yee, Matthew Welz, Maziyar Kalani, Matthew Neal

**Affiliations:** ^1^Department of Rehabilitation, Mayo Clinic, Phoenix, AZ, USA; ^2^Department of Neurological Surgery, Mayo Clinic, Phoenix, AZ, USA; ^3^Precision Neuro-therapeutics Innovation Lab, Mayo Clinic, Phoenix, AZ, USA; ^4^Neurosurgery Simulation and Innovation Lab, Mayo Clinic, Phoenix, AZ, USA

## Abstract

Lumbar spondylolysis is a unilateral or bilateral defect of the pars interarticularis, an isthmus of bone connecting the superior and inferior facet surfaces in the lumbar spine at a given level. Spondylolysis is common in young athletes participating in sports, particularly those requiring repetitive hyperextension movements. The majority of young athletes are able to return to full sport participation following accurate diagnosis and conservative management, including a structured treatment program. Surgical intervention for isolated pars injuries is seldom necessary. A progressive physical therapy (PT) program is an important component of recovery after sustaining an acute pars fracture. However, there is a paucity of literature detailing PT programs specific to spondylolysis. Here, we provide an overview of the epidemiology, natural history, radiographic evaluation, and management of pars fractures in young athletes. In addition, a detailed description of a physiotherapy program for this population that was developed at a spine center within an academic medical center is provided.

## 1. Introduction

**Figure 1:**
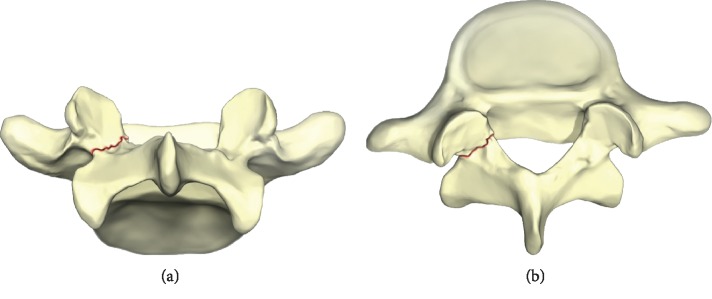
Fracture in the part of the vertebra representing spondylolysis.

Spondylolysis is a unilateral or bilateral defect of the pars interarticularis, an isthmus of bone connecting the superior and inferior facet surfaces in the spine at a given level (Figure 1). The pars defect may either be an acute fracture or a chronic osseous disruption with sclerotic borders. The condition, which has an incidence of 3–10% in the general population [1], exists on a continuum ranging from pars stress fractures to spondylolisthesis [2]. Those caring for pediatric and young adult patients participating in sports will often encounter spondylolysis which typically presents as low back pain with specific activity (i.e., lumbar extension/rotation during throwing, lumbar hyperextension, and loading during back handspring). The majority of young athletes are able to return to full sport participation following accurate diagnosis and conservative management, including a structured treatment program. Therefore, surgical intervention for isolated pars injuries is seldom necessary. A progressive physical therapy (PT) program is an important component of recovery after sustaining an acute pars fracture. However, there is a paucity of literature detailing PT programs specific to spondylolysis. Here, we provide an overview of the epidemiology, natural history, radiographic evaluation, and management of pars fractures in young athletes. Additionally, we will focus on nonoperative management, providing a detailed description of a physiotherapy program for this population that was developed at a spine center within an academic medical center.

## 2. Epidemiology and Natural History

**Figure 2:**
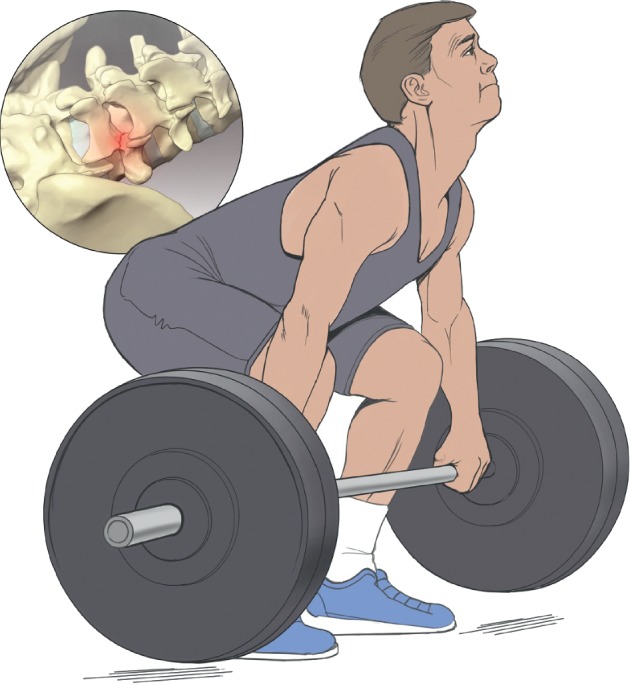
Pars fractures in athletes.

There are congenital factors such as spina bifida occulta and exaggerated lumbar lordosis that may predispose individuals to pars fractures [3]. In addition to congenital factors, activity-related exposures such as sport participation may contribute to pars fractures. Participation in sports, particularly sports involving repetitive hyperextension and axial loading of the spine, predisposes young athletes to pars fractures. Common examples include baseball, gymnastics, football, tennis, and weightlifting (Figure 2) [3, 4].

The incidence of lumbar spondylolysis in the general population is 3–10% [1]. In a radiographic study using computed tomography (CT) in 532 patients aged eight or younger presenting with general lumbar complaints, spondylolysis was diagnosed in 4.7% of the children. [1] The prevalence of lumbar spondylolysis in 153 pediatric patients, defined as younger than 19 years, who presented to an orthopedic clinic with low back pain for greater than two weeks was 39.7% [5]. All positive diagnoses of spondylolysis had a history of athletic participation. A similar study also found spondylolysis rates as high as 30% among 1025 adolescent athletes presenting to a sports medicine clinic for low back pain [6]. Thus, there is a significantly higher incidence and prevalence of spondylolysis among young athletes than those in the general pediatric population.

A review of the natural history of lumbar spondylolysis by the Scoliosis Research Society's Evidence-Based Medicine Committee (SRS EBMC) found that unilateral pars fractures typically heal normally, especially among acute fractures [7]. However, cases with bilateral and/or chronic pars fractures commonly progress to spondylolisthesis with rates as high as 43–74% [7]. Several studies have radiographically documented slip progression in populations with spondylolysis and spondylolisthesis, reporting a decreased rate of slip progression after age 20 [8–10]. It is hypothesized that ossification of the growth plates in the lumbar vertebrae helps resist anterior shear forces experienced by the aging spine [11].

In the short-term following injury, typically defined as six weeks, the majority of patients with spondylolysis will symptomatically improve, and the majority of athletes return to usual sporting activity [7]. One meta-analysis found that 92.2% of adolescent athletes who were treated conservatively and 90.3% who underwent operative intervention were able to return to athletic competition [12].

## 3. Radiographic Diagnosis

The definitive diagnosis of spondylolysis cannot be made with physical examination alone and, therefore, requires imaging for diagnosis. Various studies have evaluated one modality in comparison to a control; however, there is no absolute gold standard for radiographic diagnosis [13]. Typically, plain film x-rays are used for initial diagnosis as they are inexpensive and relatively low in radiation dosage. Although four-view plain film x-rays, which include oblique views, can be helpful in diagnosis of spondylolysis, the sensitivity is comparable to two-view films [14]. Compared to four-view x-rays, two-view films involve one-half the radiation dosage [14] and nearly two-thirds the cost [13, 14].

Advanced imaging modalities can be more sensitive than plain film x-rays in the diagnosis of spondylolysis. In comparing MRI with CT, where CT is used as the gold standard for evaluation, the sensitivity of MRI was 83% [15]. Two studies have compared CT to single-proton emission CT (SPECT) as the gold standard; they demonstrate the sensitivity of CT is 85% [16, 17]. Lastly, comparing MRI to SPECT, where SPECT was the control gold standard, the sensitivity of MRI is 80% [18].

In analyzing the appropriate diagnostic imaging algorithm, various sequences are reviewed in the literature [13, 18]. The most up-to-date systematic review of 10 major pediatric imaging studies conditionally recommends the following: MRI as the initial advanced imaging option after plain films with CT scans recommended in equivocal situations [13, 19]. MRI remains a better diagnostic tool for the early detection of pars injury than CT; however, CT is superior for longitudinal follow-up [20, 21]. CT and MRI are comparable to SPECT with significantly lower irradiation [13]. MRI more consistently diagnosed subsequent lysis than positive SPECT alone [15]. Moreover, SPECT is associated with a higher false-positive rate and correlates poorly with subsequent lysis found on other imaging modalities [16, 18]. SPECT can be helpful to identify a stress reaction in the bone that may not turn into a lysis. SPECT may be most helpful for professional athletes to define injury, stage of healing, and prognosis. Timing of initial injury may be relevant in the utilization of the imaging modality of choice. Interestingly, as conservative management and activity modification serve as primary treatment in the vast majority of pediatric spondylolysis, some question the need for X-rays or advanced imaging upfront as it does not change initial treatment [14].

In terms of cost of imaging evaluation, a 2013 study evaluated two-view and four-view plain films to CT, bone scan, and MRI. Costs grow multiplicatively from plain film imaging. Two-view and four-view plain films were estimated to cost $350 and $500, respectively; more advanced imaging costs significantly more. Bone scan ($1650), CT ($2000), and MRI ($2900) were significantly more expensive. One can assumes these numbers are higher when accounting for inflation and rising healthcare costs [14].

Our preferred algorithm is as follows: after physical examination, two-view plain films are obtained, and conservative management with activity restriction is recommended. Our advanced imaging modality of choice is MRI in an effort to reduce pediatric radiation exposure. We reserve advanced imaging for longitudinal follow-up in patients that are refractory to conservative treatment.

## 4. Surgical Treatment

The vast majority of patients with stress fractures of the lumbar pars interarticularis can be managed successfully without surgery. Kurd et al. retrospectively reviewed the medical records of 436 juvenile and adolescent patients with symptomatic spondylolysis confirmed by SPECT or CT [22]. All patients were treated nonoperatively with prescription for a thoracolumbar orthosis and discontinuation of sporting activity for three months followed by a structured PT program. At the final clinical follow-up, 95% of patients in the study achieved excellent results with the remaining individuals reporting good results and occasional use of NSAIDs. All patients returned to their preinjury level of activity, and zero patients required surgery. In 2010, Watkins and Watkins reported their 25-year experience caring for athletes with spine-related, sports injuries, utilizing a “trunk-stabilizing program.” During that period, only two athletes required operative intervention for spondylolysis [3].

In the small percentage of young patients who fail conservative treatments, direct surgical repair of the spondylolysis has been shown to be highly effective. Reports on direct surgical repair of spondylolysis focus on patients younger than 30 years of age with recommended age cutoffs for improved surgical outcomes stated as 20 or 25 years [23–25]. Criteria that should be met for direct repair of the pars include as follows: (1) healthy appearance and preserved height of the intervertebral disk on MRI and (2) no demonstration of significant motion between vertebral bodies on dynamic radiographs.

**Figure 3:**
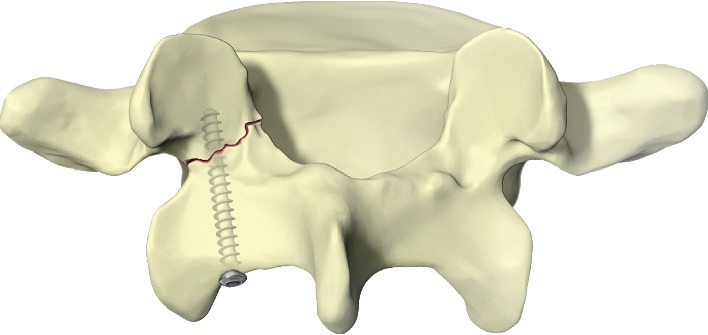
Lag screw technique.

In 1968, Kimura described bone grafting without internal fixation for the treatment of spondylolytic defects [26]. In the same time period, Scott detailed a wiring technique to augment the bone grafting of the lytic defect [27]. In 1970, Buck described a technique of placing a lag screw across the lytic defect to internally reduce the fracture (Figure 3) [28]. Numerous additional techniques have been elaborated using combinations of bone screws, wiring, hooks, and rods to immobilize and compress across the pars fracture sites. Descriptions of the individual techniques and the available outcome data are well summarized in the 2011 review article by Drazin et al., in which they conclude that any of the described techniques for repair will likely yield acceptable results in young athletes [4]. Modern minimally invasive techniques for direct spondylolysis repair may have superior clinical outcomes over conventional open techniques and may be considered in cases where conservative management has failed [29].

## 5. Nonsurgical Management of Spondylolysis

PT participation after the diagnosis of spondylolysis in young athletes is an important component of recovery in order to re-establish strength, provide education, and reintroduce patients back to sports activity. In this population, ample evidence exists to support the development of core-strengthening exercises, specifically activating and isolating the transverse abdominis (TA), internal oblique (IO), and multifidi to structurally stabilize the lumbar segments [30–32]. These deep abdominal muscles (TA and IO) and lumbar multifidus are particularly relevant in the management of patients with low back pain and/or lumbar instability because of their impact on increasing intra-abdominal pressure as well as their cocontraction resulting in tension placed on the thoracolumbar fascia and lumbar vertebrae, thereby increasing lumbar stiffness [30]. Recommendations related to the parameters of PT intervention after spondylolysis diagnosis (i.e., stage of recovery to initiate, frequency, and duration) vary significantly, resulting in a lack of standardization of postinjury rehabilitation programs. The timing of therapy initiation discussed in the literature spans from early within the diagnostic process to waiting until complete fracture healing. [2, 3] We encourage initiation of regular PT participation immediately in cases where diagnosis is suspected or confirmed.

Conflicting recommendations exist relating to the use of external bracing in the setting of spondylolysis, including timing of bracing after diagnosis. However, specific to the diagnosis of spondylolysis, there is good evidence for conservative management, including the restriction of activity/sport combined with PT and implementation of external bracing only if symptoms persist [2, 3, 33]. In a retrospective study of 121 adolescents who underwent external bracing after initial diagnosis, Selhorst et al. identified several factors that failed to show a statistically significant, long-term effect on prognosis. These factors included bracing, type of injury (unilateral, bilateral, and spondylolisthesis), duration of symptoms, and previous episode of low back pain (LBP) [34]. We do not advocate for routine external bracing in this population.

Understanding of the physiological healing process, timing of treatment intervention, appropriate progression, and graduated application of loads to the healing structure are essential for successful outcomes in any therapeutic intervention. When developing a treatment plan for a young athlete, the physical therapist should also consider other factors related to this age group including physical and emotional maturity (i.e., ability to understand concepts of care, exercise compliance) and social factors (i.e., family/social support, visit compliance). In the following portion of the paper, we will outline our recommendations for therapy including evaluation considerations, specific exercises, and exercise progression.

### 5.1. Evaluation

A thorough subjective history is an essential component of the assessment. In most states, a parent or legal guardian must provide verbal and/or written consent to allow for the treatment of a minor. They can also be a valuable source of information to fully understand the patient's medical and surgical history, psychosocial factors, previous activity, and current functional level. Psychosocial factors can impact rehabilitation outcomes, and therapists should assess patient's beliefs surrounding pain, maladaptive behaviors (avoidance or lack of fear), cultural factors, and lifestyle choices (sleeping and eating habits) [35]. Athletes can experience alienation, frustration, anger, and depression and can result in being a central driver of their pain [36].

When caring for athletic patients, we recommend documenting a detailed description of the requirements of the individual's sport including the following: type of activity, hours in practice, level of competition, conditioning routine and intensity, and additional training related to performance. Identifying whether the athlete is involved in sports seasonally or year-round is another crucial factor to consider. Most importantly, gathering specifics on provocative movements or activities performed during sports participation will prove valuable when developing a plan for treatment intervention and goal-setting.

Outcome measures utilized in the younger population with low back pain include both pain and functional scales. The visual analog pain scale (VAS) and Wong-Baker FACES are both reliable measures of subjective pain in the pediatric population and are simple tools to utilize during exercise sessions [37]. The linear analog scale of function (LAS) and the Patient-Specific Functional Scale (PSFS) are commonly utilized scales to measure activity limitation and functional outcomes. For pediatric patients, we recommend the Micheli Function Scale (MFS) at assessment and reassessments as it has been validated for assessing pain and functional levels specifically in the young athlete [38, 39]. Test and retest consistency is a key for accurate measurement when utilizing any outcome tool.

It is important for the physical examination to address all aspects of patient presentation. Adolescents and young adults with the diagnosis of spondylolysis are likely to have a variety of symptoms specific to the bony injury, secondary or compensatory symptoms, postural abnormalities, and movement dysfunction. They may develop habitual movements and postures as a result of functional activity or sport demands that may contribute to LBP. Based on the work of Sahrmann et al., “the conceptual basis of movement system impairment syndromes is that alignment in a nonideal position and repeated movements in a specific direction are thought to be associated with several musculoskeletal conditions[40]”. We believe utilizing Sahrmann's Kinesiopathologic Model of Movement System (KPM) is beneficial when treating athletes with spondylolysis. It categorizes and breaks down specific movement patterns, with lumbar extension and/or lumbar rotation being particularly relevant in this population. In addition, the KPM takes into account the interdependence of the musculoskeletal, nervous, and cardiopulmonary/endocrine systems in patient assessment. It also builds treatment directly off of assessment/reassessment of painful, aberrant, or poorly controlled motions resulting in tissue adaptation, microtrauma progressing to macrotrauma, and eventually pain [40].

Tightness of the proximal hip musculature (specifically hamstrings and hip flexors), increased lumbar muscle tone, painful spine active range of motion (AROM), and inhibition of the lumbopelvic girdle are all frequently recognized symptoms in patients diagnosed with spondylolysis. Recovery is dynamic by nature and oftentimes occurs at an accelerated pace in the young athlete. Frequent reassessment of objective measures, provocative maneuvers, and the patient's subjective complaints (stiffness, pain, and spasms) throughout care is essential to capture change and modify treatments appropriately.

Assessment and documentation of the following are recommended in the physical examination:

- Observation: posture, spinal alignment, AROM, quadrant motion, and presence of directional preference
- Gait: walking and running (if indicated)
- Palpation: lumbar muscle tenderness and tone, bony landmarks, and step-off deformity of the spine
- Movement analysis: provocative movements during sport, function, play, and activity (measure consistency with AROM limitations or directional preference); presence of aberrant movements
- Lower extremity flexibility: hamstring length and hip flexor tightness
- Manual muscle testing: gluteal musculature, hamstring, abdominal/trunk strength, and lumbar extensor endurance (if tolerable)
- Neurological evaluation: reflexes, dermatomal sensory loss, and myotomal strength loss (e.g., L5 – hip abduction, ankle dorsiflexion, and great toe extension)
- Motor control: ability to contract transverse abdominus and internal oblique

Poor clinical utility has been found for spondylolysis special testing, for example, the one-legged hyperextension test [41, 42]. Therefore, we suggest utilizing provocative movements and pain-provoking activities to guide care and re-examinations. In addition to a musculoskeletal evaluation, it is the standard of practice for clinicians to rule out any other systems that may be contributing to the patient's low back pain symptoms.

### 5.2. Early Treatment

In the acute phase, the main focus areas of intervention include (1) pain management and (2) patient education. Resolution of pain and normalized ROM are the initial goals of treatment and are utilized as a benchmark throughout the rehabilitation process to allow for progression of activity [43]. Activity, including therapeutic exercise, causing increased pain from baseline during or after the event should be discontinued or modified until improvement in symptoms occurs. At our facility, we work closely with referring physicians who will utilize NSAIDS and, in some cases, spinal injections to reduce pain initially after diagnosis to allow for improved tolerance of therapeutic activity.

Patient education should include the following:

- Eliminating specific movements, actions, or sport activities that provoke pain are the initial steps of treatment strategy [3]. Ensure that the patient understands the implications of nonhealing or worsening of the fracture.
- Demonstrate provocative maneuvers (i.e., extension and combined extension/rotation) in a way that is practical and understandable for the patient. Practice modification strategies that allow the patient to experience movement without symptom provocation in-session.
- Initiate an exercise program and ensure that the patient understands the reasoning behind each exercise.
- Revisit VAS/FACES throughout the sessions, allowing the patient to appropriately identify aggravating factors and pain management strategies without causing fear or anxiety. If the patient is highly pain-focused and excessively cautious, emphasis on pain level should be minimized.

### 5.3. Therapeutic Exercise Progression

We outline an exercise progression that we believe is comprehensive for the pediatric and young adult patient diagnosed with spondylolysis which is as follows. In addition, a three-level example of home exercise instruction is included for guidance. Please take the following into consideration:

- Independent of time after diagnosis or previous interventions (prior PT, athletic training, bracing, etc.), we recommend that the patient be introduced or reintroduced to the Level 1 routine specifically for neuromuscular re-education and to confirm that the patient is performing the routine correctly. This is an important consideration in young athletes as their bodies change rapidly and require greater emphasis on adapting to changes during growth periods.
- Prior to advancement through exercise levels, the patient must demonstrate all of the following: (1) no provocation of pain with current level exercise performance, (2) normalized AROM within activity set, and (3) successful completion of previous level's exercise requirements.
- Utilization of a tool to provide biofeedback has been recommended to assist in objective and accurate measurement of patient technique throughout treatment and as a guide for exercise progression. [30, 31].

#### 5.3.1. Level 1

**Figure 4:**
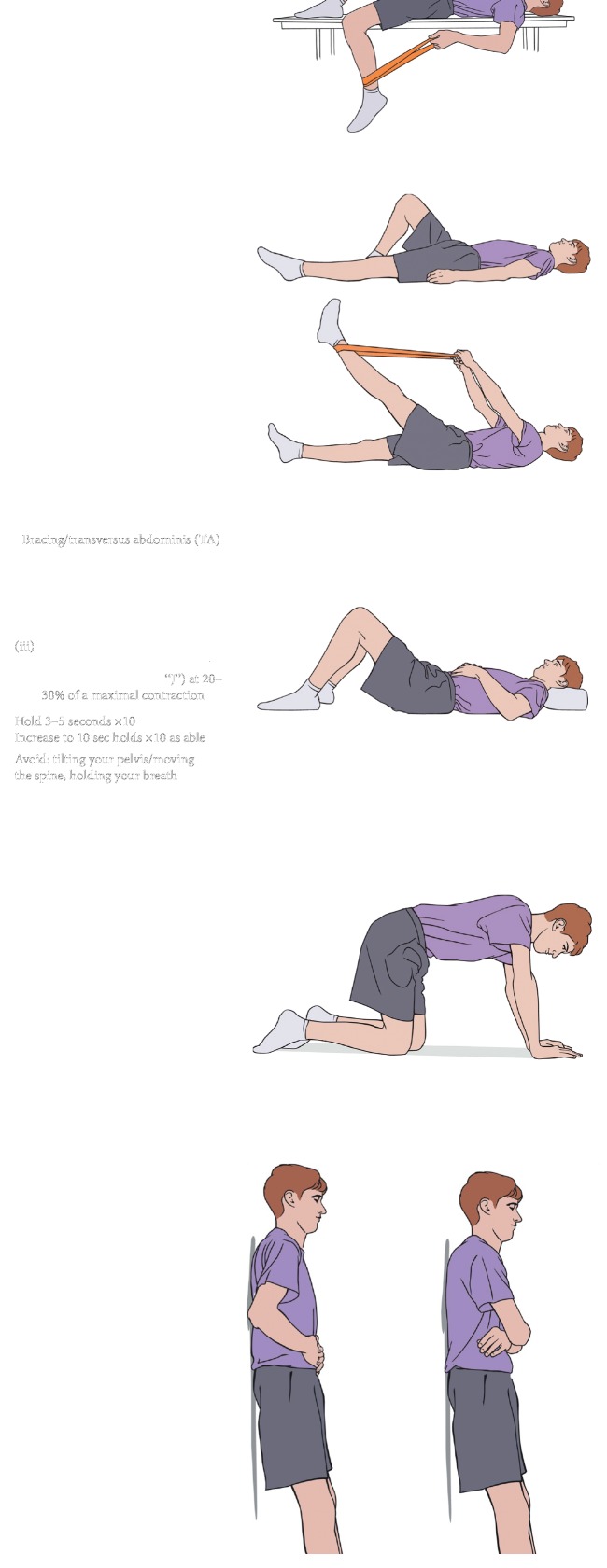
Phase 1: spondylolysis therapy rehabilitation.

(i) Strengthening/neuromuscular control: introduction to isometric TA activation in a comfortable posture, typically supine, and then progressing to variations of static posture with continued isolated TA activation without symptoms (Figure 4).
(ii) Precautions: avoid any lumbar range into provocative maneuvers. Avoid compensation from the lumbar spine, gluteals, or hamstrings when isolating the TA.
(iii) Stretch: hamstrings, hip flexors, and hip rotators to onset of resistance without pelvic compensation.
(iv) Cardiovascular: aquatic and/or land walking and stationary cycling.
(v) Conditions for transition to Level 2:
  - No increase in symptoms
  - Successful isolated TA and multifidi isometric contraction in all postures for 10 repetitions  ×10 second holds

#### 5.3.2. Level 2

**Figure 5:**
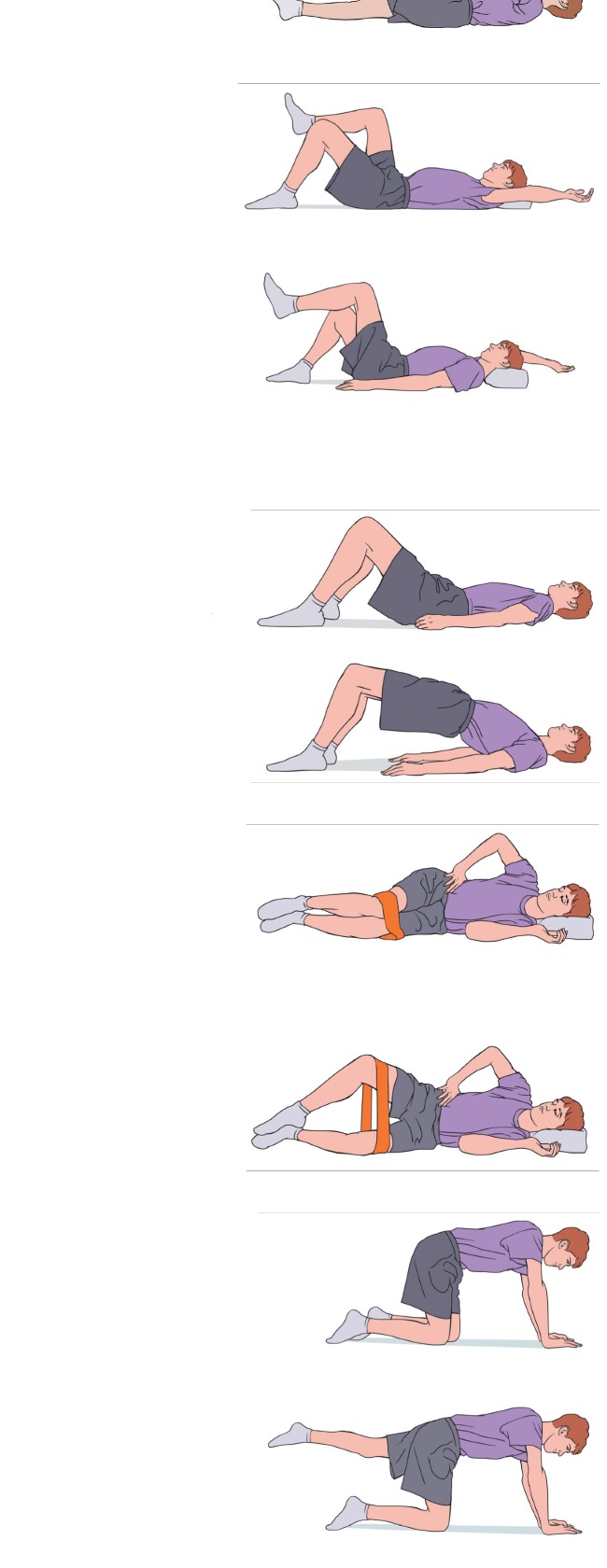
Phase 2: spondylolysis therapy rehabilitation.

(i) Strengthening/neuromuscular control: add upper extremity (UE) and lower extremity (LE) dynamic work to isolated TA/multifidi contraction. Exercises that are easily measured with biofeedback tool are recommended to carry-over from a static to a dynamic routine. Progress to standing LE and UE body-weight resistance with continued incorporation of techniques for lumbopelvic control utilized in lower level exercises (Figure 5).
  - Biofeedback tool is in the form of a pressure monitor that is mildly inflated and placed on the lumbar spine with patient supine, seated, or standing against the wall. Appropriate cocontraction of TA and multifidus can be identified by maintenance of pressure with dynamic movements of the UE and LE, indicating spinal stability with distal movement.
(ii) Stretch: hamstrings, hip flexors, and hip rotators into resistance without noted pelvic compensation.
(iii) Cardiovascular: increase the time and intensity of walking, as well as stationary cycling. Cardiovascular exercise should remain low-impact in this phase.
(iv) Conditions for transition to Level 3:
  - No increase in symptoms
  - Successful isolated TA/multifidi isometric contraction during dynamic movement
  - Ability to demonstrate 10 repetitions of a full unsupported squat to 90 degrees of knee flexion with good technique

#### 5.3.3. Level 3

**Figure 6:**
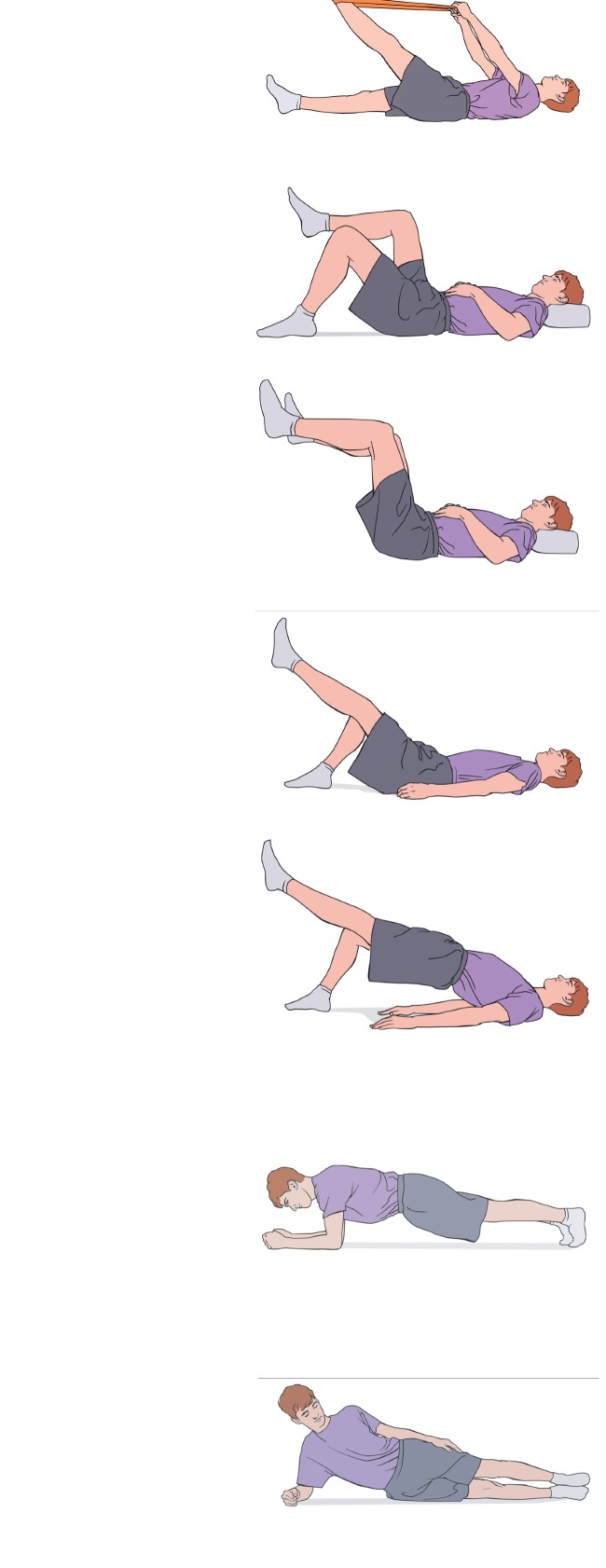
Phase 3: spondylolysis therapy rehabilitation.

(i) Progress lumbar range of motion to normal limits without symptoms (Figure 6).
(ii) Strengthening: add single-limb and unsupported hip and core exercises to previously double-limb supported activity. Continue with addition of dynamic movements throughout postures.
(iii) Stretch: resolution of the hamstring and hip flexor tone should be achieved in this phase as well as normalization of hip AROM symmetry and tolerance for end range stretching without compensation at the pelvis.
(iv) Cardiovascular: 30 minutes of walking and progression to cardiovascular exercise equipment.
(v) Conditions for transition to Level 4:
  - Successful isolation of TA and multifidi activation during asymptomatic dynamic movement
  - Elimination of directional preference with single-plane AROM

#### 5.3.4. Level 4

(i) Isolated progressing to multiplanar AROM required for the sport.
(ii) Strengthening: add variable surfaces to double-limb exercises followed by progression to single-limb activities.
(iii) Neuromuscular control: initiate low-impact, single-direction reaction time activities.
(iv) Stretch: continue with previously described hip routine. Addition of lumbar stretches if needed to achieve pain-free AROM.
(v) Cardiovascular: progress to 40 minutes of elliptical, StairMaster/VersaClimber.
(vi) Conditions for transition to Level 5:
  - No provocation of symptoms with daily activities or exercise
  - 50% return to sport practice under PT supervision

#### 5.3.5. Level 5

(i) Strengthening/neuromuscular control: progress to combined movements and coordinated range required for the sport. Agility training in multiple directions. Address specific actions that cause fear or hesitation with dynamic tasks.
(ii) Stretching and cardiovascular activities should be at practice levels.
(iii) Conditions for transition to Level 6:
  - Minimal to no supervision required during the PT home program
  - Successful return to 100% sport practice under PT supervision

#### 5.3.6. Level 6

- Patient returns to competitive sport without restriction. Continue exercise program independently. Contact medical team (PT, physician, and trainer) immediately if symptoms return.

### 5.4. Additional Considerations for Return to Sport

- Core stabilization translation to sport: TA activation and core support will need to be continuously tested, progressed, and applied to ensure sports-specific carry-over prior to full resumption of previous activity.
- Growth periods: young athletes will invariably undergo growth during and after treatment. Consistently revisiting the basic TA routine is essential to maintain the contractility and responsiveness of this muscle during periods of growth and progression of sport level.
- Overtraining: patients, parents, and coaches benefit from education on what constitutes “overtraining.” Limitations, guidelines, and recommendations specific to sport (i.e., pitch count) should be clearly communicated in order to avoid recurrence or worsening of symptoms. Overtraining should always be a consideration/concern in single-sport or year-round athletes.

## 6. Conclusion

Stress injuries and fractures to the lumbar pars interarticularis are common sports-related injuries among young athletes. After diagnosis, an individualized PT rehabilitation program including education, activity modification, and a progressive course of specific exercise will lead to symptom resolution and return to athletic participation in the vast majority of young athletes. Surgery for direct pars repair is rarely necessary; however, it has high success rates when required in refractory patients. A standardized nonoperative treatment algorithm for pediatric spondylolysis is outlined above.
